# Cholangiocarcinoma, sequential chemotherapy, and prognostic tests

**DOI:** 10.3389/fonc.2024.1361420

**Published:** 2024-06-24

**Authors:** Howard W. Bruckner, Robert De Jager, Elisheva Knopf, Fred Bassali, Abe Book, Daniel Gurell, Van Nghiem, Myron Schwartz, Azriel Hirschfeld

**Affiliations:** ^1^ Department of Oncology, MZB Foundation for Cancer Research, New York, NY, United States; ^2^ Charles E. Schmidt College of Medicine, Florida Atlantic University, Boca Raton, FL, United States; ^3^ Department of Radiology, University Diagnostic Imaging, Bronx, NY, United States; ^4^ Department of Surgery, Mount Sinai Hospital, New York, NY, United States

**Keywords:** cholangiocarcinoma, low-dosage, chemotherapy, prognosis, survival, resistance, geriatrics

## Abstract

**Introduction:**

Routine blood tests are prognostic tests for patients with cholangiocarcinoma. New drug regimens may produce a median overall survival of 2 years or more.

**Methods:**

This single practice, IRB-approved, phase II trial examines prognostic tests, Kaplan-Meier survival, and univariate Cox regression analyses. Eligibility requires: intent-to-treat; signed consent; advanced measurable intrahepatic cholangiocarcinoma, with or without resistance to the test drugs; any adult age; performance status 0–2; and expected survival of ≥ 6 weeks. Biweekly treatment, with 1/3 of standard dosages in mg/M^2^, includes: Gemcitabine 500; 5-Fluorouracil 1200 over 24 hours; Leucovorin 180; Irinotecan 80; and on day 2, Oxaliplatin 40. On progression, drugs are added on day 2: first, Docetaxel 25 precedes Oxaliplatin, with or without Mitomycin C 6 after Oxaliplatin. The next sequential additions are day 1, Cetuximab 400 total mg, then 200 mg weekly, and then Bevacizumab 10 mg/kg is substituted for Cetuximab (FDA IND# 119005).

**Results:**

For 35 patients, 19 with 1–2 lines of prior therapy, resistant tumors, and 16 no prior therapy, survival at 24-months is ≥ 72 and ≥ 58%, respectively. For 14 patients aged ≥ 70 years, ≥ 63% survive 24 months, P = 0.28. Validated tests that predict ≤ 6-month survivals find median survival times of 17-months through > 2-years when compared to patients with favorable tests: Neutrophils lymphocyte ratio > 3.0, HR = 6.54, P < 6.4x10–3; absolute neutrophil count > 8000/μl, HR = 4.95, P < 6.5x10–3; serum albumin < 3.5 g/dl, HR = 4.10, P < 0.03; and lymphocyte monocyte ratio< 2.1, HR = 1.6, P = 0.50. Overall, the 76 (60–90)% of patients with 0–2 out of 4 high risk tests survive ≥ 24 months, (P = 7.1x10–3). Treatments produce neither hospitalization, neutropenic fever, severe enteritis, nor severe neuropathies.

**Conclusion:**

Two-year survival is replicable and predictable. Findings warrant phase III validation tests of sequential regimens, re-challenge with recombination, low dosages, and blood tests that are associated with lethal mechanisms that impair response and survival.

## 1 Introduction

Cholangiocarcinoma (CCA) is projected to become the 9th leading cause of cancer-related deaths worldwide (1). Current chemotherapy for CCA results in median survival times (MSTs) of 10.5 (6.4–14.7) and 5.3 (4.1–6.6) months for primary therapy, gemcitabine and cis platin, and a mix of secondary treatments, respectively (2, 3). A phase III trial of a FOLFOX regimen found second-line MSTs of 7.2 (6.5–8.9) months and a 25.9% rate of 12-month survivors (4). The new, phase III, standard of primary care chemo-immunotherapy, with gemcitabine-cisplatin and durvalumab produces an MST of 12.9 months and 2-year survival of 23.6% (5, 6). Investigation of second-line therapy has identified 2–10 promising forms of targeted therapy, applicable to about 40% of patients. MSTs are 21.7 months for futibatinib, 21.1 months for pemigatinib, FGFR inhibitors, and 10.8 months for ivosidenib, an IDH inhibitor (4, 7–9).

Intrahepatic CCA (IHCCA), distal bile duct cancer, and gallbladder cancer (GBC) may be distinct entities; patients with IHCCA may sometimes have MSTs of 12–14 months. Management of CCA continues to present with unresolved multidisciplinary options that includes supportive care, surgery, resection to transplant, genomic evaluations, interventional radiology, radiotherapy, regional therapy, chemotherapy, targeted therapy, and chemo-immunotherapy (3–5).

Sequential test regimens have shown promising results. In previous studies, 99 patients with stage IV CCA, many of whom had resistant tumors, achieved an overall MST of more than 3 years. Added chemotherapy and targeted therapy as part of the sequence extended the patients’ MSTs by approximately 10 (6–18) months (10, 11). Derivative sequential regimens have also shown improvement in MSTs, reaching 14.5 months in an analysis of a combined group of patients, 35with CCA, 53 with new advanced pancreatic cancer (APC), 53 with resistant APC (RAPC), and 50 with resistant third-line colorectal cancers (RCRC) (12).

The use of low dosages and dose modifications of all cytotoxins prolonged survival, minimized complication rates, and avoided hospitalization. Rates of long or limiting delays of treatment were < 5% (10, 11). In contrast, standard low or high dosages of chemotherapy with 2–3 drugs alone and with added immunotherapy results in a 20–40% and 47% rate of clinically significant adverse events (AEs), respectively (2–6).

The combination of reproducible exceptional survival and safety for patients with CCA warrants further analysis of these patients’ prognostic blood tests (PBTs). An overall evaluation of parallel trials found that PBTs identified patients who may benefit from treatment (12). Also, initial PBTs have identified high and low risk subgroups of short and long survivors, among the participants in registration trials of first-line treatment for advanced CCA. PBTs were independent surrogate markers that serve as a summary variables to improve predictions of survival in comparison to clinical characteristics alone (13–16).

For patients with CCA and primary treatment, an A.L.A.N. Score (AS) model was found: where patients with 0, 1–2, or 3–4 out of 4 “unfavorable” high risk test groups had MSTs of 22, 12, and ~ 5 months, respectively (15). The 71% of patients with a favorable, low risk Neutrophil-Lymphocyte Ratio (NLR) < 3.0 had an MST of 10.6 months (P < 0.001). With an NLR of ≥ 3.0, the MST is 6.4 months, and the 95% confidence interval (CI) is 5.0–8.2 months (15). No individual low risk nor high risk PBTs identified a group of patients with an MST of > 15 or > 9 months, respectively (13–16).

There is limited experience with either the AS or individual PBTs when patients have resistant tumors; however, both the AS and individual tests predicted the overall survival outcomes of our contemporary patients with resistant tumors who received the GFLIO regimen (12). When patients were matched based on AS, serum albumin, or NLR, the elderly and heavily treated had similar survival compared to the young and new patients, respectively (17). For patients with gastric cancer, the use of baseline PBTs may assist in the evaluation and design of trials. Tests can compare patients, evaluate stratification, predict chances of survival, and identify a new subgroup of long survivors with an ECOG performance status (PS) of 2–3 (18, 19). PBTs may serve as surrogate biomarkers because mechanisms of lethality may be modified by the number of the patients’ cells, measured with the AS and PBTs (13–17). Individual cell type, neutrophils, lymphocytes, monocytes, platelets, and their subtypes produce many cytokines and growth factors that can impact tests of immunocompetence and a broad spectrum of cancer cells’ resistance to drugs (20–26).

The development of the GFLIO treatment regimen and sequence of treatment addresses safety concerns, AEs that limit treatment, and methods to reverse or circumvent the tumors’ resistance to drugs (**Schema 1**, Part A). Laboratory tests found combinations of two drugs that exhibit synergism and reverse drug resistance with inhibitory concentrations (IC) of 12 (6–25), compared to single drugs (10–12, 27). The use of low dosages can also avoid potential drug-drug antagonism observed at higher concentrations and AEs which limit treatment (28). These features allow for the simultaneous use of novel and multiple combinations of > 3 drugs (11, 12, 27–30). Independent investigations have confirmed that the simultaneous use of many combinations of two drugs is sometimes necessary in order to address the heterogeneity of tumors (31).

**Schema 1:**
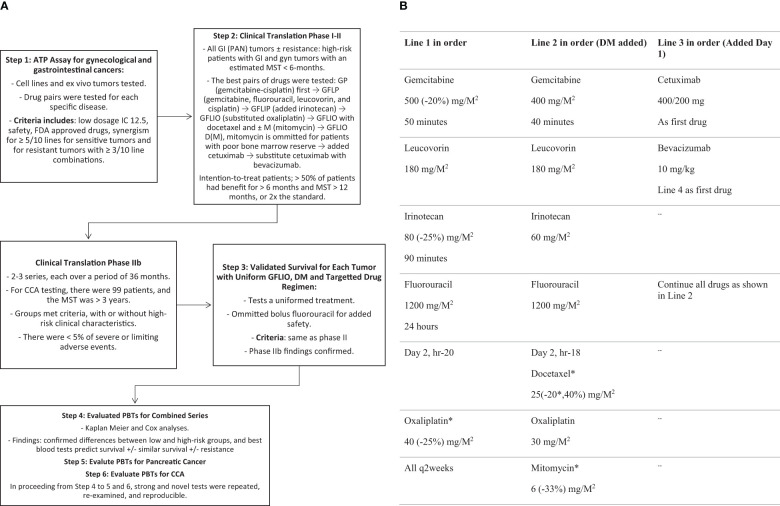
**(A)** Trial Design – Development of GFLIO ± DM ± Target Drugs. **(B)** Palliative Regimen. - Advanced Cholangiocarcinoma Palliative Regimen: All q2 weeks; -Parenthesis (): reference dosage reduction as needed for safety. - Bevacizumab replaces cetuximab at the time of progression. - Granulocyte Colony Stimulating Factor was added (300 mcg) on days 7 and 8 or 15 and 16 if the nadir neutrophil count is or will be <1000/µl or remains < 1250/µl, respectively.

These drug combinations may have other mechanisms of action. They improve immune function tests, increase the immunogenicity of RCRC, and produce response rates that reach nearly 66% (32–34). Metronomic therapy produces MSTs of 19.7 months for patients with stages 2–4 APCs, with or without prior therapy, when treatment consists of fluorouracil, oxaliplatin, and nab-paclitaxel (35). Simultaneous use of multiple drugs with other possible mechanisms of action can also be effective in patients with HIV, HTN, and hematologic cancers (36).

The prospective objectives of the analyses are to evaluate the performance of PBTs in the context of a treatment regimen that safely prolongs survival for aged and heavily treated patients. The secondary objective is to evaluate the number and size of well-defined subgroups of PBTs with prolonged survival in order to supplement the development of GFLIO and other regimens.

## 2 Methods

### 2.1 Statistics

Kaplan-Meier, Cox, and Greenwood’s analyses examine intention-to-treat patients and estimated 2-year survival rates starting from the first day of treatment. The effects of AS and historically validated PBTs as low risk vs. high-risk groups, age, and prior therapy were estimated using log-rank and Cox proportional hazard (PH) regression analyses with a 95% CI. Statistical software included first-round analyses with a proprietary package and validation of the analyses with open access software. Validation of the analysis utilized open access software, survminer R and python packages, scikit-learn software for survival curves and seaborn for visualization of the statistical data.

### 2.2 Test parameters

Variables included prior treatment, drug resistance, age, gender (**Table 1**), AS (**Table 2**), and individual PBTs as validated high vs. low-risk groups (**Table 3**). Survival times were estimated from the first day of treatment. Ages of 60, 65, 70, 75, and 80 were tested as cutoff points. The panel of PBTs includes favorable, low risk parameters: lymphocytes > 1.5/µl; platelets < 300,000/µl; white blood count (WBC) < 10,000/µl; absolute neutrophil count (ANC) < 8,000/µl; serum albumin > 3.5 g/dl; as well as NLR < 3.0 and the LMR > 2.1. The AS is defined by the number, 0–4/4 unfavorable high-risk tests: serum albumin < 3.5 g/dl; absolute neutrophil count >8,000/µl; neutrophil lymphocyte ratio > 3.0; and lymphocyte monocyte ratio < 2.1 (15, 16).

**Table 1:** Estimated Kaplan-Meier 2-year rates of survival and Cox regression analyses for patients with no prior treatment or resistant tumors.

Variable	Number of Patients	≥ 2-year S	HR	HR ± CI	P Value
–	# (%)	%	–	95%	–
**All Patients**	35 (100)	66	–	–	–
**NPT**	16 (46)	58	0.98	0.34 – 3.07	0.97
**Resistance**	19 (54)	72	1.02		
**< 70 years**	21 (60)	67	0.55	0.61 – 5.47	0.28
**≥ 70 years**	14 (40)	63	1.82		
**Female**	17 (49)	69	0.84	0.36 – 3.88	0.77
**Male**	18 (51)	64	1.19		

Statistical tests evaluate hazard ratio (HR), 95% confidence interval (CI), and P values, comparing no prior therapy to prior therapy resistant tumors. The age cutoff was at 70 years old. Survival was measured from the first day of treatment with GFLIO. At the time of subsequent progressions, the first drugs added were docetaxel and mitomycin, followed by the added target drugs cetuximab and bevacizumab. Survival was not evaluated past 2 years.

**Table 2:** Estimated Kaplan-Meier median survival time (MST), rate of survival at 2 years, and Cox-regression analyses for 35 patients with advanced intrahepatic cholangiocarcinoma.

CC	Number of Patients	MST	2-year S	2 Year S CI	P
–	# (%)	Months	%	%	–
All	35 (100)	>24	66	48–72	–
AS 0	12* (34)	>24	90	**	0.10
AS 0–1	15 (43)	>24	82	**	0.02
AS 0–2	27 (77)	>24	**77.6**	60–90	7.1x10^-3^
AS 1–2	15 (43)	>24	66	56–76	–
AS 3–4	8* (23)	17	13	**	7.1x10^-3^

Survival is measured from the first day of treatment with GFLIO. At the time of subsequent progression, the first drugs added were Docetaxel and mitomycin, followed by the added target drugs, cetuximab and bevacizumab. Tests evaluate hazard ratio (HR), confidence interval (CI), and P value, comparing subgroups with an A.L.A.N. score (AS) of 0, 1–2, and 3–4. The AS is the number of high-risk unfavorable tests: serum albumin < 3.5 g/dl; absolute neutrophil count >8,000/µl; neutrophil lymphocyte ratio > 3.0; and lymphocyte monocyte ratio < 2.1.

- *AS 0 and 3–4 patients were measured as exploratory individual survival endpoints.

- ** CI could not be generated because the sample size was too small.

**Table 3:** Estimated Kaplan-Meier median survival time (MST), rate of survival at 2 years, and Cox regression analyses for 35 patients with advanced intrahepatic cholangiocarcinoma.

Test	AV	Number of Patients	MST	2-yr S	HR	CI	P
–	–	# (%)	Months	%	–	–	–
**NLR**	< 3	15 (43)	>24	92	0.15	1.7–25.2	6.4x10^-3^
	≥ 3	20 (57)	17	34	6.54		
**ANC**	≤ 8	25 (71)	>24	84	0.20	1.6–15.7	6.5x10^-3^
	> 8	10 (29)	17	18	4.95		
**WBC**	< 10	24 (69)	>24	84	0.22	1.4–14.4	0.01
	≥ 10	11 (31)	17	19	4.53		
**Platelets**	≤ 300	25 (71)	>24	84	0.20	1.3–17.8	0.02
	> 300	10 (29)	17	27	4.88		
**Albumin**	≥ 3.5	26 (74)	>24	72	0.24	1.2–14.4	0.03
	< 3.5	9 (26)	17	36	4.1		
**Lymph**	≥ 1.5	21 (60)	>24	68	0.50	0.6–6.56	0.27
	< 1.5	14 (40)	>24	57	1.98		
**AlP**	< 135	13 (37)	>24	92	0.63	0.5–4.79	0.42
	≥ 135	22 (63)	22.1	49	1.58		
**LMR**	≥ 2.1	25 (71)	>24	74	0.68	0.5–4.18	0.50
	< 2.1	10 (29)	22.1	47	1.6		

Statistical tests compare stratification for individual assay values (AV) with validated prognostic blood tests (PBTs). These include neutrophil lymphocyte ratio (NLR), absolute neutrophil count (ANC), white blood count (WBC), platelets, serum albumin, lymphocytes (lymph), alkaline phosphatase (AlP), and lymphocyte monocyte ratio (LMR). Statistical tests compare stratification for each individual AV as a low vs. high-risk test group, measuring the hazard ratio (HR), 95% confidence interval (CI), and P value. Survival is measured from the first day of treatment with GFLIO. At the time of subsequent progression, the first drugs added were docetaxel and mitomycin, followed by the added target drugs, cetuximab and bevacizumab.

### 2.3 Patients

This study involved a compassionate extension of an IRB-approved trial. The eligibility criteria for participation included the following: intention-to-treat; active progression of disease; advanced measurable IHCCAs and Klatskin tumors; IRB written consent; Helsinki practices (37); any adult age; PS 0–2; positive biopsy; and an anticipated survival of > 6 weeks through < 52 weeks. The patients’ tumors could either be new or resistant to active standard treatment with two or more test drugs, gemcitabine, a platin (usually cisplatin), and sometimes fluorouracil, either as a 48-hour infusion or as capecitabine.

Patients were ineligible for the study if they had metastases in the CNS; recent hospitalization or IV as treatment for dehydration within the past 2 weeks; inability to reach the office; or unresolved NCI grade 3 blood tests or grade 2 co-conditions (38). The entry of patients into the study commenced in May 2016 and was closed in May 2018. Data entry closed for analysis was completed in May 2019.

### 2.4 IRB requirements

The IRBs’ criteria for continuation of patient accrual included a combined rate of less than 10%: grade 4 hematologic and grade 3 other complications; treatment withdrawal; hospitalization, or a forced delay of more than 3 days in treatment. Real-time monitors evaluated > 50% rates of benefit as well as safety because of both the novel low dosages and entry of patients with high-risk characteristics such as age, PS, or prior limiting AEs. This study was approved by the Western IRB and an FDA application (IND #119005).

### 2.5 Treatment

The sequence (**Schema 1**, Part B) involved biweekly administration of gemcitabine (G) at a dosage of 500 mg/M^2^ over 50 minutes, then irinotecan (I) at a dosage of 80 mg/M^2^ over 90 minutes. This was followed by continuous infusions of 5-fluorouracil (F) at a dosage of 1200 mg/M^2^ over 24 hours, leucovorin (L) at a dosage of 180 mg/M^2^, and on day two, oxaliplatin (O) at a dosage of 40 mg/M^2^. This drug regimen constitutes GFLIO. Drugs were added to the regimen upon disease progression, first docetaxel (D), at a dosage of 25 mg/M^2^, with or without mitomycin C (M) at a dosage of 6 mg/M^2^, a maximum of 10 mg. Mitomycin C was omitted for 1–2 cycles when patients had a prior treatment limiting cytopenia. For a second progression, cetuximab was added at a dosage of 400 mg on day 1, and then 200 mg weekly. For a third progression, bevacizumab at a dosage of 10 mg/kg on day 1, every two weeks, replacing cetuximab. The prior drugs, except for cetuximab, were continued as part of each regimen.

### 2.6 Serial tests

Tests conducted initially and throughout the study included a CBC every week; a physical examination, a CMP every two weeks; tumor markers monthly, and computer tomography scans at six and then every 12 weeks. Tests are repeated each time a new drug is added to the regimen and to evaluate new clinical complaints (11, 12). The latter include 1 grade change in symptoms, PS, liver function tests including bilirubin, alkaline phosphatase, AST, and ALT, and within those with a PS of 2 or a worsening Karnofsky score. Progression and resistance to prior therapy is defined by both RECIST criteria and independent referring oncologists (39). Progression during treatment with GFLIO is defined with a new full battery of tests including the best prior imaging method, CT or MRI.

### 2.7 Evaluation

Data was registered prospectively in real-time and in redundant electronic databases. Reviewers included dedicated data-management staff, independent oncologists, radiologists, and statisticians. Second reviewers included oncologists who re-examined the eligibility of the patients and exclusions. There were only 2 patients excluded, one with gall bladder adenocarcinoma and one who refused treatment. Preset statistical analyses were followed by similar analyses with a second software package with the use of historical cutoffs and comparison to parallel trials. Analyses are categorized as exploratory if the sample size was less than 14 patients or if the P value was > 0.01. Exploratory analyses were selected because they found possible interactions between PBTs, age and prior therapy for the overall group of contemporary patients (12).

### 2.8 Dose modification

Treatment with the regimen could result in brief neutropenia (1500–750) or thrombocytopenia (125,000–75,000). Granulocyte colony-stimulating factor (G-CSF) 300 mcg was given for 2 days, days 7 and 8 or 15 and 16 if the ANC was < 1000/µl or < 1250/µl on those respective days. Thereafter, 2 days of G-CSF were given with every following cycle, unless the 2 dyas of G-CSF caused the ANC > 8000/µl, in which case only 1 day of G-CSF would be given on day 7. If G-CSF produced an ANC > 1250/µl, treatment continued on day 15 or 17 without changes in the dosages of the chemotherapy. Cetuximab was administered for patients with and without KRAS mutations, in contrast to practices for patients with APC or CRC (10–12, 40, 41). Neither targeted FDFR or IDG, or targeted immunotherapy was available during the study period.

Initial dosages of cytotoxins were reduced by the percentages shown in **Schema 1**, Part B, for patients with: prior grade 4 hematologic or grade 3 other AEs; cytopenia needing more than 2 days of G-CSF; fragility; PS of 2; treatment delay exceeding 7 days; or (absent in this series) nadir sepsis (11, 12). Initially, on cycle 1, oxaliplatin or mitomycin C can be omitted or the dosage of docetaxel reduced to 15 mg/M^2^ for patients with high-risk characteristics. Subsequently, the omitted drugs could be re-introduced at 66% of level 1 dosages. Dosage can be increased by 12.5% to a maximum of level 1 dosages, in the absence of grade 4 ANC or platelet counts. Bevacizumab and irinotecan were withheld in a stop-go fashion until complete resolution of grade 1–2 enteritis. Fluorouracil was escalated monthly by 20%, no more than twice, in the absence of stomatitis and enteritis. Treatment was discontinued when the PS fell to 3, if rest or supportive care failed to improve the PS.

## 3 Results

**Figure 1:**
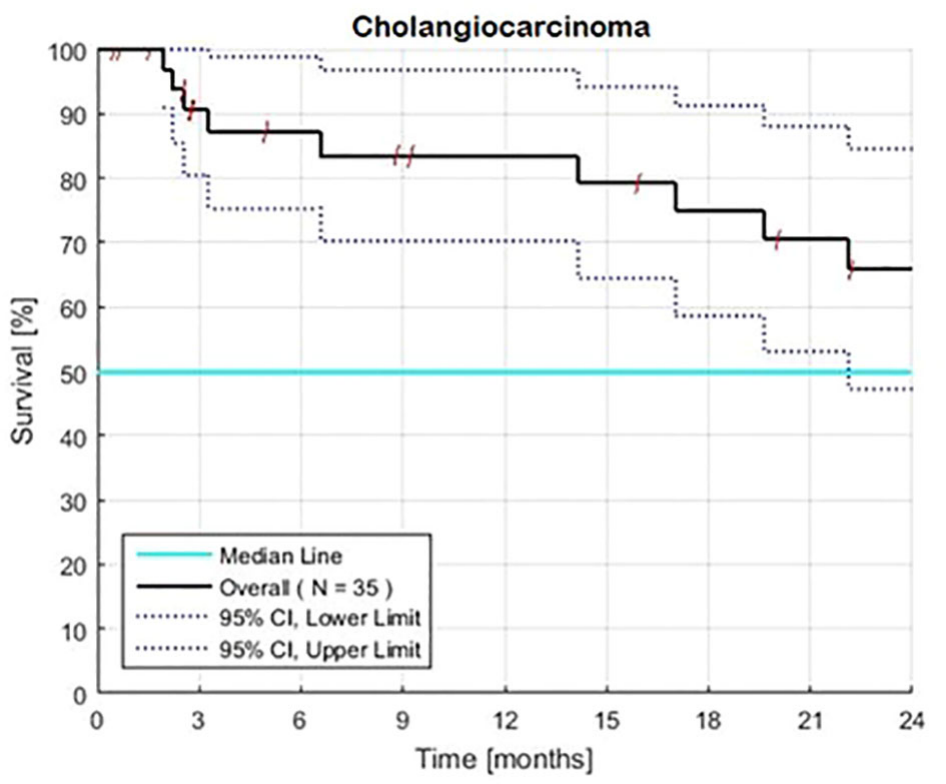
Estimated Kaplan-Meier overall survival of 35 patients with advanced intrahepatic cholangiocarcinoma with (N=19) and without (N=16) prior therapy. The overall 2-year rate of survival is 66% and the associated 95% confidence interval is 48–85%. Survival was calculated from the first day of treatment with GFLIO. At the time of progression, docetaxel/mitomycin C were added, and then subsequently cetuximab and then bevacizumab.

Overall survival rates for 35 patients, including 19 with resistant and 16 with new, no prior chemotherapy were 88, 80, and 62% at 12, 18, and 24 months (**Table 1**, **Figure 1**). The 95% CI for the lower limit of overall MST was 21.5 months. The 95% CI for the rates of overall survival at 12 and 24 months were 70–97% and 48–85%, respectively.

### 3.1 Gender and age

**Figure 2:**
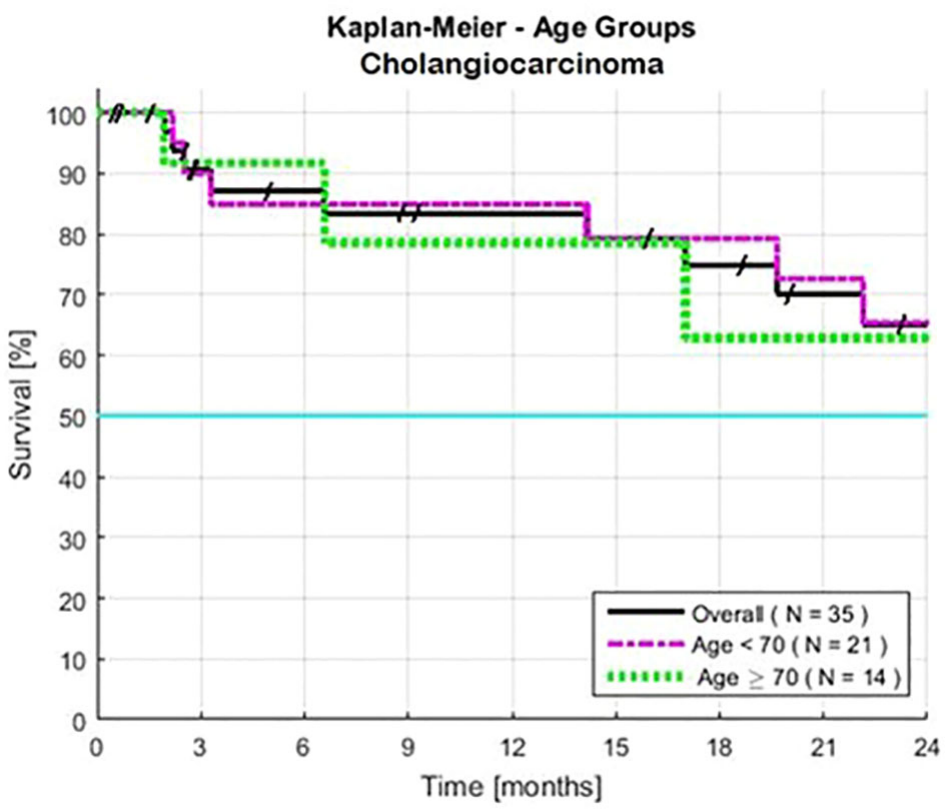
Estimated Kaplan-Meier and the impact of age for patients with advanced intrahepatic cholangiocarcinoma. Cutoff points were ≥ 70 years old (N=14), and < 70 years old (N=21). Two-year survivals were similar, >24 months for both subsets. Survival was calculated from the first day of treatment with GFLIO. At the time of progression, docetaxel/mitomycin C were added, and then subsequently cetuximab and then bevacizumab.

Both males and females had similar survival rates (**Table 1**). Survival rates were also similar for patients of all ages (P = 0.28) (**Table 1**, **Figure 2**). Among patients aged 70 or older (40% of the total), the HR was 1.82. An exploratory analysis of 36 months finds a modest trend, an advantage for the young compared to the elderly patients (not shown).

### 3.2 Treatment history

**Figure 3:**
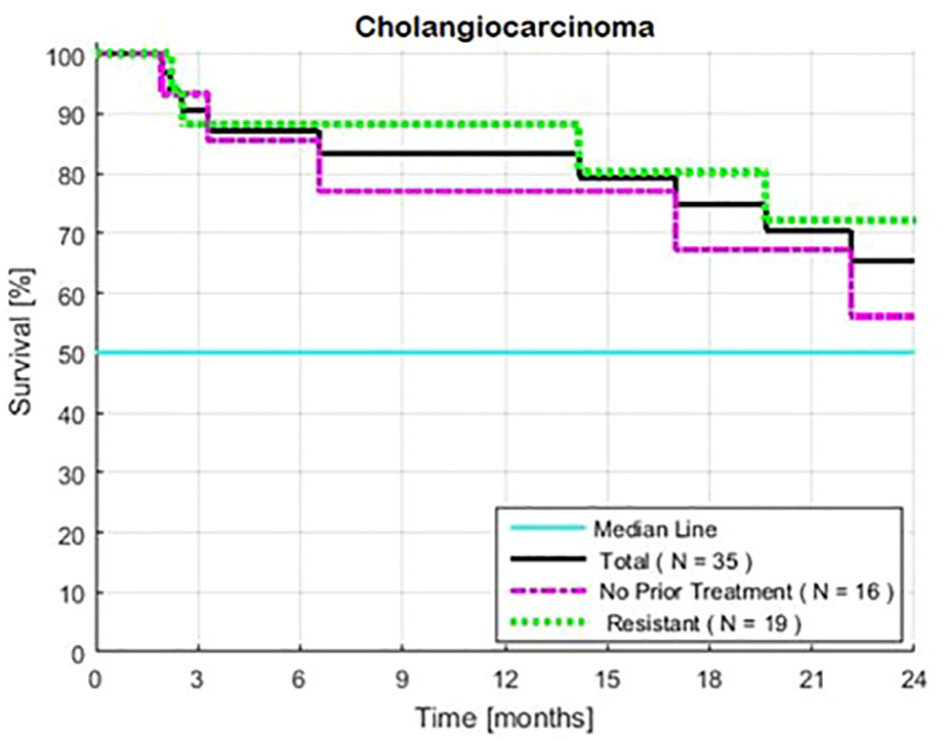
Estimated Kaplan-Meier and the impact of treatment (with or without 1–2 prior lines of treatment for patients with intrahepatic cholangiocarcinoma). Two-year rates of survival with and without prior treatment were 72 and 56%, respectively. Survival was calculated from the first day of treatment with GFLIO. At the time of progression, docetaxel/mitomycin C were added, and then subsequently cetuximab and then bevacizumab.

Survival was similar for the patients with or without prior standard therapy (HR = 1.02, P = 0.97) (**Table 1**, **Figure 3**). The frequency of low-risk tests were similar in both groups.

### 3.3 A.L.A.N. scores

**Figure 4:**
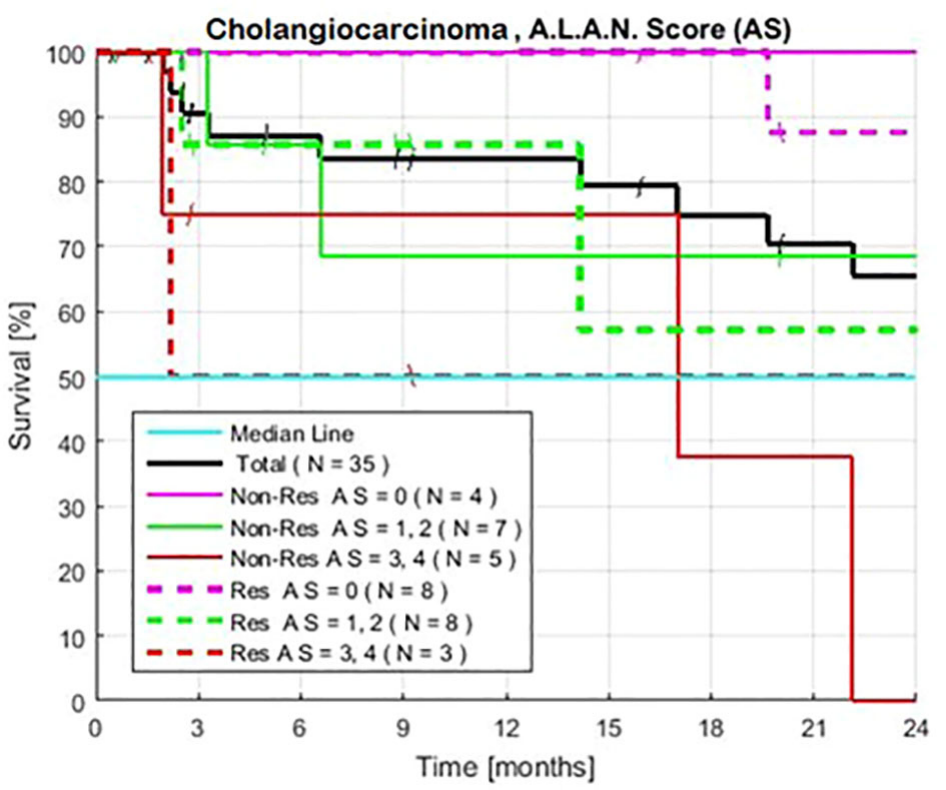
Exploratory estimated Kaplan-Meier survival., without and without prior treatment when patients are defined by the A.L.A.N. Score of 0, 1–2, and 3–4, the number of historically unfavorable tests: serum albumin < 3.5 g/dl; absolute neutrophil count >8,000/µl; neutrophil lymphocyte ratio > 3.0; and lymphocyte monocyte ratio < 2.1. Observed survival is similar for both groups of patients with matched AS.

Patients with an AS of 0 (no high-risk tests) (N = 12) had survival rates of 100%, 100%, and 90% at 12, 18, and 24 months, respectively (**Table 2**, **Figure 4**). Patients with an AS of 1–2 high-risk tests (N = 15) had survival rates of 76%, 66%, and 66% (CI 56–76%). Among patients with an AS of 0–2 (N = 27), 76% (CI 60–90%) survived beyond 24 months. Patients with an AS of 3–4 unfavorable tests (N = 8) had survival rates of 67, 34, and 13%. A comparison of patients with an AS of 0–2 vs. 3–4 showed an HR of 6.29 and a P value of 7x10^-3^.

### 3.4 Prognostic blood tests

Individual PBTs, in their descending order of statistical strength: NLR, ANC, WBC, platelet count, and serum albumin (**Table 3**). Groups with low-risk tests have 2-year survivals ranging from 72–92%. Groups with high-risk tests had MSTs ranging from 17 to ≥ 24 months. High-risk tests predict 2-year survival rates for high NLR,34%; high alkaline phosphates,49%; low serum albumin,36%; low lymphocytes,57%; and low LMR,47%. Tests differ in their order of statistical strength as well as their associated median and 2-year survival compared to the combined series (12).

### 3.5 Prior therapy

Matched groups of patients with an AS of 0 and 3–4, with and without prior therapy, showed similar long and short exploratory survival rates (**Figure 4**). Survival was also similar for patients with an AS of 1–2, with or without prior therapy, when these patients had similar NLR, ANC, or serum albumin levels, as observed in the combined series of patients (12).

### 3.6 Safety

There are neither hospitalizations, neutropenic fever, nor novel AEs due to chemotherapy. The rates of grade 1–3 AEs did not show clinically significant changes compared to reported experiences with standard therapy (2, 4).

## 4 Discussion

The sequential treatments replicate the MST of > 24 months in patients with IHCCA (8). Each of four PBTs (NLR, ANC, WBC, and serum albumin) divide patients into statistically significant low and high-risk groups in the expected fashion. When PBTs are the same, survival tends to be similar for both the young and elderly patients. As a new observation, it is also nearly equal for patients with or without prior therapy (12, 42–44).

Novel features of the treatment include integration of: re-challenge; continued and re-introduction of drugs; recombination with and without the addition of new synergistic drugs; simultaneous use of four or more drugs; low dosages; all reverse or bypass the tumors’ resistance to critical drugs (12). These findings, along with promising similar evidence of efficacy in parallel series support and expand upon the findings of investigations of the individual features of the treatment (12, 42, 45).

Evaluation of this series of patients with CCA finds that PBTs may independently modify and improve investigation of eligibility, small groups of patients, and mechanisms of resistance or lethality. PBTs can be screening surrogate biomarkers for inflammation, driver growth factors, cytokines, and immunosuppression (12–16, 20–26).

The prolonged survival in many low and high-risk test groups when compared to historical test groups indicates that the treatment contributes to improved survival within the known limitations of cross-trial comparisons. High-risk AS tests alone may not be a contraindication for the treatment of individual patients with CCA (16). Patients with high-risk tests can be included in trials, especially if there is a prospective plan to evaluate the PBTs and high-risk clinical characteristics in order to avoid false-negative conclusions (14). Such findings are consistent for patients with 1–2 high-risk tests; however, practice for patients with 3–4 high risk tests requires caution because patients with some other cancers have little or no benefit.

To the best of our knowledge, no combination of standard therapy and clinical characteristics can identify a group of stage IV patients with MSTs similar to those observed in this group with an AS of 0 or an AS of 1–2, or the similar prolonged MSTs compared to the expected survival of patients with APC,RAPC, and RCRC (7).

Some combination of the sequential treatments and an AS of 0–2 as eligibility criteria may minimize the poor safety outcomes resulting from age, frailty, maximum tolerated dosage, and resistance to drugs. These characteristics limit the real-world eligibility and utilization of clinically valuable drugs for the majority of patients with advanced GI cancers (2, 3, 42–44).

The use of low dosage is a promising additional approach in order to expand indications and for the development of drugs (35, 45, 46). Treatment with the regimen has shown no novel and exceptionally few occurrences of grade 4 or limiting AEs, although idiopathic and outlier events remain possible at low dosage. The slow decline of the nadir ANCs over multiple cycles allows for dose adjustments or the addition of minimal yet rapidly acting G-CSF to avoid prolonged cytopenia. Since 2020, when individual baseline PBTs are severely unfavorable, *ad hoc* exploratory practice includes both early recognition of relapse or impending crises, and addition of docetaxel and mitomycin (DM), with or without targeted therapy, to increase the chances of rapid response. Also, our anecdotal findings and independent work have produced long lasting response with the combinations of GFLIO-DM or single drugs, respectively, after failure of both the individual treatments with chemotherapy and immunotherapy (32–34, 47, 48). However, the patients in this series did not receive immunotherapy.

Limitations of the study are the small number of patients and the underpowered subgroups with high-risk tests. Other limitations are the absence of: a randomized comparator; multivariate tests to confirm the independence of PBTs and clinical characteristics; re-examination of our promising multi-disease experience with DM, bevacizumab, or cetuximab as sequentially added drugs (10, 11, 13–16, 49, 50).

Individual drugs may sometimes require the synergistic effects produced with the novel addition of individually ineffective irinotecan, docetaxel, or mitomycin. Target drugs may also inhibit tumor drivers that become clinically important only after multiple lines of treatment. Development efforts require both validation and caution, because the added drugs, as well as nab paclitaxel, docetaxel and liposomal irinotecan, have all shown limited or inconsistent effectiveness (51–55). Independent laboratory investigations have also found promising, sometimes broad synergy between mitomycin and the individual drugs, including gemcitabine, fluorouracil, irinotecan, oxaliplatin, and docetaxel drugs (56).

The atypical high P values of the groups with high-risk LMR and absolute lymphocyte count (ALC) in analyses of both our CCA and combined series of patients compared to earlier investigations suggests that the treatments improve the patients’ immune function.

The prolonged survival of groups with favorable tests may be evidence of unintended patient selection in referral practices. Nevertheless, the number of patients in statistically powerful subgroups with an AS of 0–2 allows for investigation of personalized PBTs both in exploratory retrospective fashion and as comparator tests for future phase III trials.

## 5 Conclusion

Many lines of evidence support our findings and meet the criteria for undertaking phase II-III comparisons of new and standard regimens. Survival is reproducible overall and in what may be surrogate biomarker subgroups, novel for many unfavorable subgroups, and applicable to patients with resistant tumors both in this and the large APC combined analyses (12, 41). The regimens integrate promising exploitable and infrequently examined features, safe dosages for combination chemotherapy, with or without laboratory assistance, broadly applicable and clinically promising methods to bypass resistance to drugs, improve safety of palliative care, and restore the patients’ immune functions. It also develops rechallenge-recombination, synergism with immunotherapy, target drugs, and analogues of the cytotoxins (4).

PBTs warrant investigation as supplemental comparators of treatments and as eligibility criteria to personalize the order, timely addition of drugs, and identify targetable lethal mechanisms. Addressing these objectives can benefit the majority of patients with CCA because they have historically short survival, resistant tumors, new options for immunotherapy, and concerns related to safety because of advanced age, and now avoidable high rates of AEs (31).

## Data availability statement

The raw data supporting the conclusions of this article will be made available by the authors, without undue reservation.

## Ethics statement

The studies involving humans were approved by Western Institutional Review Board. The studies were conducted in accordance with the local legislation and institutional requirements. The participants provided their written informed consent to participate in this study.

## Author contributions

HB: Conceptualization, Data curation, Funding acquisition, Investigation, Methodology, Project administration, Resources, Supervision, Validation, Visualization, Writing – original draft, Writing – review & editing. RD: Validation, Writing – original draft, Writing – review & editing. EK: Validation, Writing – original draft, Writing – review & editing. FB: Formal analysis, Resources, Software, Writing – review & editing. AB: Data curation, Formal analysis, Writing – original draft. DG: Resources, Supervision, Writing – original draft. VN: Data curation, Formal analysis, Writing – original draft. MS: Formal analysis, Investigation, Project administration, Resources, Writing – original draft. AH: Conceptualization, Investigation, Methodology, Project administration, Resources, Supervision, Writing – original draft.
